# Left ventricular diastolic volume on cardiac magnetic resonance and risk of incident heart failure

**DOI:** 10.1093/ehjopen/oeag009

**Published:** 2026-01-24

**Authors:** Julio Núñez, Gema Miñana, Miguel Lorenzo, Rafael de la Espriella, Agustín Fernández-Cisnal, Nerea Perez, Elena De Dios, Jose Gavara, Cesar Rios-Navarro, Victor Marcos-Garces, Hector Merenciano, Juan Sanchis, Antoni Bayés-Genís, Markus Meyer, Vicent Bodí

**Affiliations:** Cardiology Department, Hospital Clínico Universitario de Valencia, Avenida Blasco Ibáñez 17, Valencia CP 46010, Spain; Instituto de Investigación Sanitaria INCLIVA, Calle Menéndez y Pelayo, 4, Valencia 46010, Spain; Centro de Investigación Biomédica en Red - Cardiovascular (CIBER-CV), Calle Monforte de Lemos, 3-5, Pabellón 11, Madrid 28029, Spain; Department of Medicine, School of Medicine and Odontology, University of Valencia, Avenida de Blasco Ibáñez, 15, Valencia 46010, Spain; Cardiology Department, Hospital Clínico Universitario de Valencia, Avenida Blasco Ibáñez 17, Valencia CP 46010, Spain; Instituto de Investigación Sanitaria INCLIVA, Calle Menéndez y Pelayo, 4, Valencia 46010, Spain; Centro de Investigación Biomédica en Red - Cardiovascular (CIBER-CV), Calle Monforte de Lemos, 3-5, Pabellón 11, Madrid 28029, Spain; Department of Medicine, School of Medicine and Odontology, University of Valencia, Avenida de Blasco Ibáñez, 15, Valencia 46010, Spain; Cardiology Department, Hospital Clínico Universitario de Valencia, Avenida Blasco Ibáñez 17, Valencia CP 46010, Spain; Instituto de Investigación Sanitaria INCLIVA, Calle Menéndez y Pelayo, 4, Valencia 46010, Spain; Cardiology Department, Hospital Clínico Universitario de Valencia, Avenida Blasco Ibáñez 17, Valencia CP 46010, Spain; Instituto de Investigación Sanitaria INCLIVA, Calle Menéndez y Pelayo, 4, Valencia 46010, Spain; Cardiology Department, Hospital Clínico Universitario de Valencia, Avenida Blasco Ibáñez 17, Valencia CP 46010, Spain; Instituto de Investigación Sanitaria INCLIVA, Calle Menéndez y Pelayo, 4, Valencia 46010, Spain; Instituto de Investigación Sanitaria INCLIVA, Calle Menéndez y Pelayo, 4, Valencia 46010, Spain; Centro de Investigación Biomédica en Red - Cardiovascular (CIBER-CV), Calle Monforte de Lemos, 3-5, Pabellón 11, Madrid 28029, Spain; Instituto de Investigación Sanitaria INCLIVA, Calle Menéndez y Pelayo, 4, Valencia 46010, Spain; Instituto de Investigación Sanitaria INCLIVA, Calle Menéndez y Pelayo, 4, Valencia 46010, Spain; Department of Medicine, School of Medicine and Odontology, University of Valencia, Avenida de Blasco Ibáñez, 15, Valencia 46010, Spain; Cardiology Department, Hospital Clínico Universitario de Valencia, Avenida Blasco Ibáñez 17, Valencia CP 46010, Spain; Instituto de Investigación Sanitaria INCLIVA, Calle Menéndez y Pelayo, 4, Valencia 46010, Spain; Centro de Investigación Biomédica en Red - Cardiovascular (CIBER-CV), Calle Monforte de Lemos, 3-5, Pabellón 11, Madrid 28029, Spain; Cardiology Department, Hospital Clínico Universitario de Valencia, Avenida Blasco Ibáñez 17, Valencia CP 46010, Spain; Instituto de Investigación Sanitaria INCLIVA, Calle Menéndez y Pelayo, 4, Valencia 46010, Spain; Centro de Investigación Biomédica en Red - Cardiovascular (CIBER-CV), Calle Monforte de Lemos, 3-5, Pabellón 11, Madrid 28029, Spain; Cardiology Department, Hospital Clínico Universitario de Valencia, Avenida Blasco Ibáñez 17, Valencia CP 46010, Spain; Instituto de Investigación Sanitaria INCLIVA, Calle Menéndez y Pelayo, 4, Valencia 46010, Spain; Centro de Investigación Biomédica en Red - Cardiovascular (CIBER-CV), Calle Monforte de Lemos, 3-5, Pabellón 11, Madrid 28029, Spain; Department of Medicine, School of Medicine and Odontology, University of Valencia, Avenida de Blasco Ibáñez, 15, Valencia 46010, Spain; Cardiology Department, Hospital Universitari Germans Trias I Pujol, Carretera de Canyet, s/n, 08916 Badalona, Barcelona, Spain; Department of Medicine, Lillehei Heart Institute, University of Minnesota College of Medicine, 2231 6th Street SE, Suite 4-156 Minneapolis, MN 55455, USA; Cardiology Department, Hospital Clínico Universitario de Valencia, Avenida Blasco Ibáñez 17, Valencia CP 46010, Spain; Instituto de Investigación Sanitaria INCLIVA, Calle Menéndez y Pelayo, 4, Valencia 46010, Spain; Centro de Investigación Biomédica en Red - Cardiovascular (CIBER-CV), Calle Monforte de Lemos, 3-5, Pabellón 11, Madrid 28029, Spain; Department of Medicine, School of Medicine and Odontology, University of Valencia, Avenida de Blasco Ibáñez, 15, Valencia 46010, Spain

**Keywords:** Left ventricle volumes, Heart failure, Cardiac magnetic resonance, Sex, Left ventricle ejection fraction

## Abstract

**Aims:**

We explored the association between indexed left ventricular end-diastolic volume (iLVEDV) on cardiac magnetic resonance (CMR) and the risk of incident heart failure (HF) (stage C) in patients with suspected coronary heart disease without overt HF (stages A and B). We also examined the risk-modifying effect of left ventricular ejection fraction (LVEF) and sex.

**Methods and results:**

We retrospectively included 5471 patients who underwent vasodilator stress CMR for suspected coronary artery disease and without a history of HF. Multivariate Cox proportional hazards regression adapted for competing events models assessed the relationship between iLVEDV and new-onset HF (stage C), considering LVEF status as a potential modifier. The mean age was 651 ± 11.6 years, and 2123 (38.8%) were women. The medians of CMR-iLVEDV and CMR-LVEF were 67 mL/m^2^ (55–80) and 66% (58–72), respectively. At a median (p25% to p75%) follow-up of 5.1 years (2.3–8.2), we registered 287 new-onset HF diagnosis. iLVEDV was associated with a U-shaped risk of incident HF. The risk was most pronounced with an iLVEDV > 100 mL/m^2^ and <45 mL/m^2^. Notably, the association between iLVEDV and HF risk was influenced by LVEF status. In patients with LVEF < 50%, a higher risk was found in those with larger iLVEDV. On the contrary, LVEF > 60%, lower iLVEDV identified those at increased risk.

**Conclusion:**

In subjects with higher LVEF, smaller left ventricle volumes identified a subset of patients with an increased risk of incident HF. These findings reveal a need for a better understanding of the pathophysiological mechanisms of HF with supranormal ejection fraction.

## Graphical Abstract

**Graphical Abstract:**
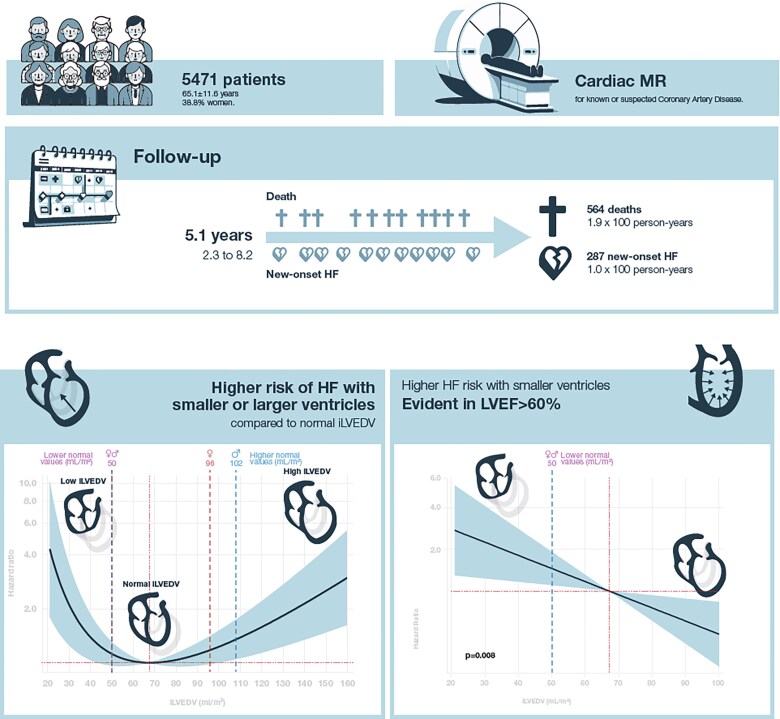
Left ventricular diastolic volume and risk of new-onset HF. HF: heart failure; iLVEDV: indexed left ventricle end-diastolic volume; LVEF: left ventricle ejection fraction; CMR: magnetic resonance.

## Introduction

Enlarged left ventricular volumes are associated with a higher risk of heart failure (HF), mainly when systolic dysfunction is present.^1^ Recent findings suggest that a smaller left ventricular dimension is associated with lower aerobic capacity in healthy women with normal systolic function.^2^ Along this line, some studies indicate that HF with supranormal ejection fraction is more common in women with smaller ventricular dimensions, greater diastolic dysfunction, and increased sympathetic tone.^2,3^ In the general population, higher left ventricular ejection fraction (LVEF) has been reported to be associated with a higher risk of adverse outcomes, particularly among individuals with a lower stroke volume (SV).^4^ Thus, we hypothesized that increased ventricular contractility may be a compensatory mechanism aiming to maintain SV in those with smaller left ventricular volumes. Consequently, smaller left ventricular dimensions combined with higher LVEF may identify individuals with limited cardiac output reserve, potentially predisposing them to the onset of clinical HF.

This study aimed to assess the association between lower left ventricular end-diastolic volume and the risk of new-onset HF (stage C) and whether this association is influenced by LVEF status.

## Methods

### Study sample

This study constitutes a retrospective examination of data from a comprehensive prospective registry encompassing 6675 consecutive patients referred for vasodilator stress cardiovascular magnetic resonance (CMR) examinations due to known or suspected coronary artery disease (CAD). In patients with previously documented CAD, the indication for CMR was the presence of new or worsening symptoms suggestive of ischaemia, warranting reassessment. Patients with a prior diagnosis of HF, and the presence of known cardiomyopathy, infiltrative disease, or moderate-severe valvular disease were excluded. The tests were conducted at a single healthcare centre in Valencia, Spain (Departamento de Salud Clínico-Malvarrosa) from 2001 to 2016. Clinical information and CMR data were systematically documented and promptly entered into a predefined database. After excluding cases with incomplete baseline data (*n* = 84), individuals lost to follow-up (*n* = 71), incomplete CMR sequences, including missing left ventricular volumes (*n* = 622), and prior diagnosis of HF (stage C) or cardiomyopathy/valvular diseases (*n* = 427), a total of 5471 patients were included in this analysis (see Supplementary material online, *Figure S1*). Patients were classified into stages A (no structural heart disease, LVEF ≥ 50%, and no prior ischaemic heart disease) or B HF (structural heart disease or LVEF < 50%, or prior ischaemic heart disease). The investigation conforms with the principles outlined in the Declaration of Helsinki’ (Br Med J 1964; ii: 177), and all participating patients provided informed consent. In September 2018, the local ethics committee approved a retrospective analysis of adverse clinical events.

### CMR analysis

Images from 1.5 Tesla CMR were analysed using specialized software (Syngo, Siemens, Erlangen, Germany). Parameters including indexed (mL/m^2^) left ventricular end-systolic and end-diastolic volumes (iLVEDV), and LVEF were quantified from cine images. Standardized iLVEDV using the Z score (Z_iLVEDV) was also calculated using normal sex-specific reference values.^5^ Stroke volume index (SV) was also calculated [iLVEDV − indexed end-systolic volume (iLVEDSV)]. LVEF was assessed by the Simpson method and categorized into three categories: <50, 50–60, and >60%. For LVEF and indexed left ventricle (LV) volumes assessment, epicardial and endocardial borders were automatically detected in end-systolic and end-diastolic phases for all slices and then visually inspected and manually corrected if necessary. LV mass and indexed LV mass/end-diastolic volume ratio as the CMR parameter equivalent of the relative wall thickness^6^ were also registered in 4492 subjects. The extent (number of segments) showing hypokinesia, ischaemia, and late gadolinium enhancement (LGE) was visually identified using the 17-segment model. Ischaemia was defined as a segmental perfusion deficit (PD), characterized by a sustained delay (in at least three consecutive temporal images compared to other segments in the same slice) during the initial passage of contrast through the myocardium after vasodilator administration. The ischaemic burden represented the number of segments exhibiting post-stress PD. Stress-induced PD was ruled out in segments with transmural LGE and those with simultaneous PD and non-transmural LGE, where the extent of PD did not surpass that of LGE. Technical aspects of CMR studies are presented in Supplemental Methods and elsewhere.^7–10^

### Clinical endpoints

The primary endpoint was new-onset HF. Additionally, all-cause mortality, spontaneous acute myocardial infarction (AMI), and coronary revascularization were registered during follow-up. New-onset HF encompassed a new diagnosis of HF at the outpatient level or hospitalization for acute HF according to guidelines. In all cases, the diagnosis required a verified registration of HF diagnosis in the medical records and always verification of treatment with loop diuretics. Acute HF hospitalization was defined as any unscheduled inpatient stay that exceeded 24 h, requiring intravenous therapy to ameliorate HF symptoms and signs. In the total of new-onset HF diagnoses, NT-proBNP and echocardiographic LVEF were registered at 98.9% and 64.2% of patients, respectively. The number of patients with echocardiographic LVEF assessment (Echo-LVEF) within the 3 months after diagnosis was 250 (87.1%) of those with incident HF. Acute coronary syndromes (Killip class > I) were not classified as admission for acute HF. AMI was defined according to the fourth definition of myocardial infarction.^11^ Additionally, revascularization procedures related and unrelated to CMR findings were also documented. CMR-related revascularization was identified by either coronary artery bypass grafting, or percutaneous coronary intervention performed within 3 months following the index vasodilator stress CMR study, as long as no hospital admission for cardiovascular indications had taken place during that period (in this case, patients were censored upon readmission). The assessment of outcomes was performed by reviewing electronic medical records (EMRs) of the public health care system in the Valencian Community. The follow-up and adjudication of clinical endpoints utilized data from the SIA-GAIA and Orion Clinics EMRs, which comprehensively record all medical interactions occurring in the public healthcare system of the Valencian Community. Clinical follow-up was centrally adjudicated by four clinical cardiologists authorized by the local ethics committee, utilizing the unified regional EMR. The cardiologists in charge of endpoint ascertainment were blinded to CMR data at baseline.

### Statistical analysis

Continuous variables are presented as mean (± standard deviation) or median (p25% to p75%). Categorical variables are expressed as percentages. Baseline variables were compared across iLVEDV quintiles using the ANOVA or Kruskal–Wallis tests (continuous) or χ^2^ (discrete) test.

Using the Fine and Gray method, the association of iLVEDV with time-to-new onset clinical HF was assessed using multivariate Cox proportional hazards regression models accounting for the effect of competing events.^12^ All-cause mortality, MI, and non-related CMR revascularization during follow-up were considered competing events. All covariates shown in *Table 1* were evaluated for predictive purposes. The covariates included in the multivariate models were selected based on their biological/clinical plausibility, regardless of the *P*-value (see Supplementary material online, *Table S1*). The linearity assumption for all continuous variables was simultaneously tested and transformed, if appropriate, with fractional polynomials.^13^ The final multivariate model included the following baseline covariates: age, sex, diabetes mellitus, hypertension, dyslipidaemia, smoking status, prior known CAD, prior revascularization, left bundle branch block, functional ability to perform an electrocardiography stress test, LV septum and posterior wall thicknesses, CMR ischaemic burden (0–17 segments), segments with LGE, and related revascularization (those occurred within 3 months following the stress CMR study). Under this multivariate setting, the association between iLVEDV across LVEF categories (<50%, 50–60%, and >60%) was evaluated. A sensibility analysis exploring the interaction between LV mass/end-diastolic volume ratio and LVEF categories (<50%, 50–60%, and >60%) was also performed.

**Table 1:** Baseline characteristics across iLVEDV quintiles

Variable	Total (*n* = 5471)	Q1 (19–53 mL/m^2^) (*n* = 1094)	Q2 (53–62 mL/m^2^) (*n* = 1094)	Q3 (62–71 mL/m^2^) (*n* = 1095)	Q4 (71–84 mL/m^2^) (*n* = 1094)	Q5 (84–240 mL/m2) (*n* = 1094)	*P*-value for trend
Age, years	65.1 ± 11.6	67.6 ± 10.3	66.5 ± 10.8	64.9 ± 11.5	63.2 ± 12.2	63.3 ± 12.5	<0.001
Sex (woman), *n* (%)	2123 (38.8)	503 (46.0)	506 (46.3)	456 (41.6)	407 (37.2)	251 (22.9)	<0.001
Hypertension, *n* (%)	3561 (65.1)	719 (65.7)	743 (67.9)	702 (64.1)	695 (63.5)	702 (64.2)	0.337
Diabetes, *n* (%)	1552 (28.4)	343 (31.4)	318 (29.1)	329 (30.0)	277 (25.3)	285 (26.1)	0.336
Dislipidaemia, *n* (%)	3131 (57.2)	661 (60.4)	644 (58.9)	613 (56.0)	597 (54.6)	616 (56.3)	0.395
Smoker, *n* (%)	986 (18.0)	183 (16.7)	191 (17.5)	204 (18.6)	181 (16.5)	227 (20.7)	0.164
BMI, kg/m^2^	28.7 ± 8.0	29.8 ± 11.9	28.6 ± 4.2	28.5 ± 4.4	28.5 ± 10.6	28.1 ± 5.4	<0.001
Previous revascularization, *n* (%)	961 (17.6)	143 (13.1)	190 (17.4)	180 (16.4)	208 (19.0)	240 (21.9)	0.093
Cerebrovascular disease, *n* (%)	138 (2.5)	22 (2.0)	35 (3.2)	24 (2.2)	18 (1.6)	39 (3.6)	0.121
Previous IHD, *n* (%)	2000 (36.6)	355 (32.4)	373 (34.1)	374 (34.2)	414 (37.8)	484 (44.2)	0.051
Previous AMI, *n* (%)	978 (17.9)	149 (13.6)	171 (15.6)	185 (16.9)	206 (18.8)	267 (24.4)	0.195
Family history of IHD, *n* (%)	291 (5.3)	44 (4.0)	50 (4.6)	55 (5.0)	70 (6.4)	72 (6.6)	0.902
Stage A HF, *n* (%)	2415 (44.1)	474 (43.3)	538 (49.2)	555 (50.7)	518 (47.3)	330 (30.1)	<0.001
Left BBB, *n* (%)	308 (5.6)	35 (3.2)	41 (3.7)	56 (5.1)	64 (5.9)	112 (10.2)	0.019
ST-segment depression, *n* (%)	158 (2.9)	21 (1.9)	33 (3.0)	30 (2.7)	35 (3.2)	39 (3.6)	0.737
CMR LVEF, %	64.26 ± 11.0	69.96 ± 8.4	68.16 ± 8.5	66.16 ± 9.1	63.46 ± 9.6	53.76 ± 11.3	<0.001
iLVEDV, mL/m^2^	69.6 ± 21.5	44.6 ± 6.1	57.7 ± 2 .8	66.6 ± 2.5	77.1 ± 3.7	102.1 ± 19.2	<0.001
iLVESV, mL/m^2^	26.2 ± 15.6	13.5 ± 4.3	18.4 ± 5.1	22.7 ± 6.2	28.2 ± 7.8	48.3 ± 18.4	<0.001
Septum, mm	11.7 ± 2.7	12.2 ± 2.6	11.7 ± 2.7	11.5 ± 2.7	11.4 ± 2.6	11.7 ± 2.8	<0.001
Posterior wall, mm	8.5 ± 1.9	9.0 ± 2.0	8.6 ± 1.9	8.4 ± 1.9	8.3 ± 1.9	8.4 ± 1.9	<0.001
LVMI, g/m^2^	69.0 ± 18.6	59.0 ± 13.2	62.9 ± 14.4	66.2 ± 15.9	71.1 ± 15.1	85.7 ± 21.0	<0.001
LGE, segments	1.2 ± 2.1	0.6 ± 1.4	0.7 ± 1.4	0.9 ± 1.8	1.2 ± 2.0	2.4 ± 2.9	<0.001
Patients with LGE ≥ 1 segments, *n* (%)	1780 (32.5)	245 (22.4)	269 (24.6)	314 (28.7)	372 (34)	580 (53.0)	<0.001
Hypokinesia, no. of segments	1.4 ± 2.7	0.8 ± 1.7	1.0 ± 2.1	1.0 ± 2.0	1.4 ± 2.5	2.8 ± 4.1	<0.001
Ischaemic burden, no. of segments	2.1 ± 3.0	1.6 ± 2.6	1.7 ± 2.7	1.8 ± 2.7	2.1 ± 3.0	3.2 ± 3.5	<0.001
Patients with ≥1 ischaemic segments, *n* (%)	2239 (40.9)	366 (33.5)	385 (35.2)	416 (38.0)	465 (42.5)	607 (55.5)	0.001
Related revascularization, *n* (%)	486 (8.9)	91 (8.3)	86 (7.9)	96 (8.8)	100 (9.1)	113 (10.3)	0.806

AMI, acute myocardial infarction; BBB, bundle branch block; BMI, body mass index; IHD, ischaemic heart disease; LGE, late gadolinium enhancement; iLVEDV, indexed left ventricular end-diastolic volume; iLVESV, indexed left ventricular end-systolic volume; LVEF, left ventricular ejection fraction; LVMI, left ventricular mass index; PCI, percutaneous coronary intervention.

The proportionality assumption for the hazard function over time was tested using the Schoenfeld residuals. Harrell’s C-statistics evaluated the multivariate model’s discriminatory ability.

We set a two-sided *P*-value of <0.05 as the threshold for statistical significance. STATA 18.1 (STATA Statistical Software, StataCorp LP, College Station, TX, USA) was used for the analysis.

## Results

### Baseline characteristics

The mean age was 65.1 ± 11.6 years, and 2123 (38.8%) were women. Hypertension and dyslipidaemia were prevalent at 3561 (65.1%) and 3131 (57.2%), respectively. Diabetes mellitus, smoking, and AMI were present in 1552 (28.4%), 986 (18.0%), and 978 (17.9%). The mean body mass index was 28.7 ± 8, and the median of iLVEDV, LVEF, and iLVSVi were 67 mL/m^2^ (55–80), 66% (58–72), and 43 mL/m^2^ (36–50), respectively. The mean Z-iLVEDV was −0.73 ± 1.52. The proportion of patients with baseline LVEF <50%, 50–60%, and >60% was 652 (11.9%), 1061 (19.4%), and 3758 (68.7%), respectively. The median LV posterior and septal wall thicknesses were 11 mm (10–13) and 9 mm (7–9), respectively. The median indexed LV mass/end-diastolic volume ratio was 0.98 g/mL (0.82–1.19). Individuals categorized as stage A and B HF were 2415 and 3056.

Baseline characteristics across iLVEDV quintiles are presented in *Table 1*. Patients in the upper quintiles were more frequently men with higher rates of left bundle branch block and a greater CMR-ischaemic and necrosis burden. On the contrary, smaller volumes were more common in older subjects, women, and those with higher body mass index, LV wall thicknesses, LVEF, and stage A HF (*Table 1*). There was a stepwise increase in the proportion of those with LVEF >60% when moving from lower to upper iLVEDV quintiles (Q1: 31.4%, Q2: 66.9%, Q3: 75.2%, Q4: 82.6%, and Q5: 86.5%, *P* < 0.001). We did not find differences in rates of related coronary revascularizations (those that occurred within the first 3 months after CMR) among iLVEDV quintiles.

### Adverse clinical events

At a median (p25% to p75%) follow-up of 5.1 years (2.3 to 8.2), we registered 564 deaths (1.9 × 100 person-years), 287 new-onset stage C HF diagnosis (1.0 × 100 person-years), 177 AMI (0.6 × 100 person-years), and 374 non-CMR-related revascularizations (1.3 × 100 person-years). A total of 170 new-onset HF diagnoses (59.2% of the total) were due to acute HF hospitalizations. When available (*n* = 250), the median (p25% to p75%) LVEF assessed by echocardiography in patients with new-onset HF was 59% (44% to 68%). The distribution of patients according to LVEF categories was as follows: 34.8% had an LVEF <50%, 16.8% had an LVEF between 50 and 60%, and 48.4% had an LVEF >60%. When comparing these echocardiographic LVEF results to prior CMR-LVEF categories, 82.8% of patients with an echo-LVEF, <50% and 91.7% of those with an echo-LVEF, and >60% remained in the same category. For patients with an echo-LVEF between 50% and 60%, 54.7% remained in the same category.

### iLVEDV and incident stage C HF

**Figure 1:**
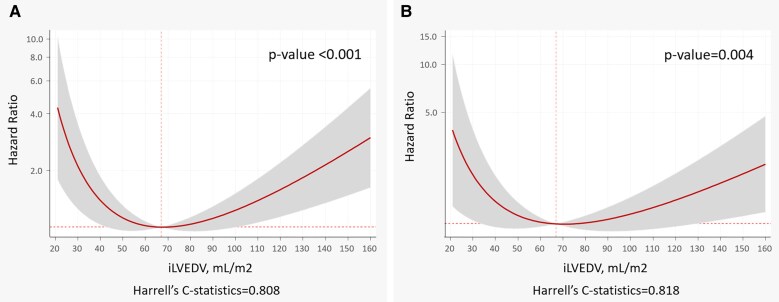
Indexed left ventricular end-diastolic volume and risk of incident HF in the whole sample. (*A*) Risk of incident HF. (*B*) Risk of HF hospitalization. iLVEDV, indexed left ventricle end-diastolic volume; HF, heart failure.

Baseline variables associated with incident HF are shown in *Table 2*. Rates of new-onset HF were more common in those in the upper (1.60 × 1.0 × 100 person-years) followed by the lowest (1.12 1.0 × 100 person-years) iLVEDV quintiles. Cumulative HF incidence plots, accounting for competing events, also showed the highest rates in those in the upper quintile, followed by subjects in the lowest (see Supplementary material online, *Figure S2*). In the whole sample, after multivariate adjustment and accounting for competing events, iLVEDV remained significantly associated with the risk of incident HF in a U-shaped pattern (*Figure 1A*, *P* < 0.001). The nadir or risk was found between 60 and 80 mL/m^2^, and a significantly increased risk was noted for iLVEDV >100 and <45 mL/m^2^ (*Figure 1A*). The c-statistic of the model was 0.808. A similar U-shaped pattern of risk was also noticed when the endpoint was acute HF hospitalizations (*Figure 1B*, *P* = 0.001). Likewise, we also found a U-shaped risk curve when only subjects on stage A (*n* = 2415) were examined (see Supplementary material online, *Figure S3*).

**Table 2:** Baseline characteristics across new-onset heart failure

Variable	Total (*n* = 5471)	No (*n* = 5184)	Yes (*n* = 287)	*P*-value
Age, years	65.1± 11.6	64.7 ± 11.6	71.3 ± 10.0	<0.001
Sex (woman), *n* (%)	2123 (38.8)	1989 (38.4)	134 (46.7)	0.005
Hypertension, *n* (%)	3561 (65.1)	3352 (64.7)	209 (72.8)	0.005
Diabetes, *n* (%)	1552 (28.4)	1410 (27.2)	142 (49.5)	<0.001
Dislipidaemia, *n* (%)	3131 (57.2)	2956 (57.0)	175 (61.0)	0.118
Smoker, *n* (%)	986 (18.0)	952 (18.4)	34 (11.8)	0.005
BMI, kg/m^2^	28.7 ± 8.0	28.7 ± 8.2	29.4 ± 4.7	0.145
Previous revascularization, *n* (%)	961 (17.6)	911 (17.6)	50 (17.4)	0.020
Cerebrovascular disease, *n* (%)	138 (2.5)	128 (2.5)	10 (3.5)	0.286
Previous IHD, *n* (%)	2000 (36.6)	1875 (36.2)	125 (43.6)	0.011
Previous AMI, *n* (%)	978 (17.9)	930 (17.9)	48 (16.7)	0.601
Family history of IHD, *n* (%)	291 (5.3)	279 (5.4)	12 (4.2)	0.378
Left BBB, *n* (%)	308 (5.6)	283 (5.5)	25 (8.7)	0.020
ST-segment depression, *n* (%)	158 (2.9)	149 (2.9)	9 (3.1)	0.797
CMR-LVEF, %	64.2 ± 11.0	64.5 ± 10.8	59.4 ± 13.4	<0.001
iLVEDV, mL	69.6 ± 21.5	69.3 ± 20.8	75.3 ± 31.1	<0.001
iLVESV, mL	26.2 ± 15.6	25.8 ± 14.8	34.0 ± 24.6	<0.001
Septum, mm	11.7 ± 2.7	11.7 ± 2.7	12.4 ± 2.9	<0.001
Posterior wall, mm	8.5 ± 1.9	8.5 ± 1.9	8.8 ± 2.0	0.012
LVMI, g/m^2^	69.0 ± 18.6	68.5 ± 18.2	78.0 ± 23.2	<0.001
LVMI/iLVEDV	1.04 ± 0.30	1.03 ± 0.30	1.10 ± 0.32	<0.001
LGE, segments^a^	1.2 ± 2.1	1.1 ± 2.1	1.6 ± 2.5	0.001
Hypokinesia, segments	1.4 ± 2.7	1.3 ± 2.6	2.4 ± 3.8	<0.001
Ischaemic burden, segments	2.1 ± 3.0	2.0 ± 2.9	3.1 ± 3.6	<0.001
Related revascularization, *n* (%)	486 (8.9)	447 (8.6)	39 (13.6)	0.004

AMI, acute myocardial infarction; BBB, bundle branch block; BMI, body mass index; IHD, ischaemic heart disease; LGE, late gadolinium enhancement; iLVEDV, indexed left ventricular end-diastolic volume; iLVESV, indexed left ventricular end-systolic volume; LVEF, left ventricular ejection fraction; LVMI, left ventricular mass index; LVMI/iLVEDV, left ventricular mass index/indexed left ventricular end-diastolic volume; PCI, percutaneous coronary intervention.

^a^Data are available in 4492 subjects.

### iLVEDV and incident stage C HF: the role of LVEF

**Figure 2:**
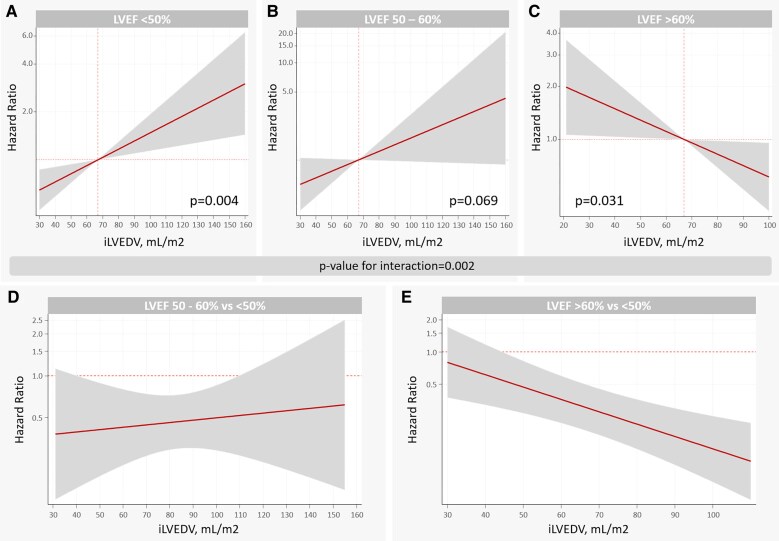
Indexed left ventricular end-diastolic volume and risk of incident HF across LVEF categories. (*A*) LVEF <50%, (*B*) LVEF 50–60%, (*C*) LVEF >60%, (*D*) LVEF 50–60% vs. < 50%, and (*E*) LVEF >60% vs. < 50%. iLVEDV, indexed left ventricle end-diastolic volume; HF, heart failure; LVEF, left ventricle ejection fraction.

**Figure 3:**
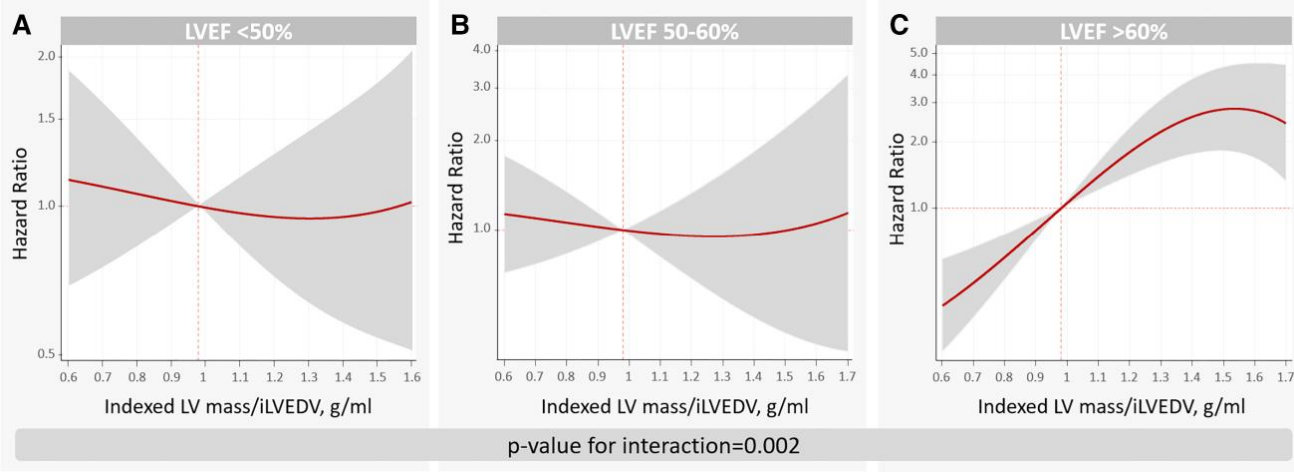
Indexed LV mass/end-diastolic volume ratio and risk of incident heart failure across LVEF categories. (*A*) LVEF <50%, (*B*) LVEF 50–60%, (*C*) LVEF >60%, LV, left ventricle; iLVEDV, indexed left ventricle end-diastolic volume; LVEF, left ventricular ejection fraction.

Cumulative incidence plots showed that bigger volumes identify those with higher rates of HF if LVEF was <50 and 50–60% (see Supplementary material online, *Figure S4*). Conversely, smaller volumes identified individuals with elevated rates of HF if the LVEF was >60% (see Supplementary material online, *Figure S4*). Further adjusted analyses confirmed that the association between iLVEDV and risk of new-onset HF was significantly modified by LVEF (*P*-value for interaction = 0.002). iLVEDV was positive and linearly associated with a higher risk of new-onset HF when LVEF <50% (*Figure 2A*). A weaker association was noted for patients with LVEF 50–60% (*Figure 2B*). On the opposite, in patients with LVEF >60%, the risk of incident HF was significantly higher in those with lower iLVEDV (*Figure 2C*). Thus, per increase in 10 mL/m^2^ of iLVEDV, the risk of HF increased by 13% in individuals with LVEF <50% (HR = 1.13, 95% CI: 1.04–1.22; *P* = 0.004). On the contrary, an increase in 10 mL/m^2^ of iLVEDV in those with LVEF >60% reduced the risk by 14% (HR = 0.86, 95% CI: 0.75–0.98; *P* = 0.031). Compared to patients with LVEF <50%, those with 50–60% showed a lower risk of HF along most of the continuum of iLVEDV (*Figure 2D*). Patients with LVEF >60% showed a lower risk of HF except for those with iLVEDV <45 mL/m^2^, in which the risk was comparable to those with LVEF <50% (*Figure 2E*). A similar differential association between iLVEDV and acute HF across LVEF categories was also found (*P*-value for interaction = 0.020), as shown in Supplementary material online, *Figure S5*. A subgroup analysis found that the differential association between iLVEDV and LVEF categories was also present regardless of limiting the evaluation to patients with stage A or B HF (see Supplementary material online, *Figure S6*). We also find a heterogeneous association of Z-iLVEDV and risk of incident stage C HF across the categories of LVEF (*P*-for interaction = 0.002). Z-iLVEDV was positively associated with the risk of new-onset HF in subjects with LVEF < 50% but inversely associated with the endpoint when LVEF > 60% (see Supplementary material online, *Figure S7*). Similar heterogeneous findings were found when the interaction between SV and LVEF categories was selected as the exposure (*P*-value for interaction = 0.022). Lower SV was associated with a higher risk of new-onset HF in those with LVEF > 60%. In the rest of the LVEF categories, SV was not significantly associated with the outcome (see Supplementary material online, *Figure S8*). In a sensitivity analysis exploring the adjusted association between indexed LV mass/end-diastolic volume ratio and risk of new-onset HF across LVEF categories (*n* = 4492), we found that this ratio was also differentially linked to the risk of incident HF (*P*-value for interaction = 0.002). In those with LVEF < 50 and 50–60%, the indexed LV mass/volume ratio was not related to the risk of the endpoint (*Figure 3A* and *B*, respectively). On the contrary, the higher ratio was linked to heightened risk in those with LVEF >60% (*Figure 3C*).

### iLVEDV and risk of incident stage C HF in subjects with LVEF > 60%: the influence of age, sex, and LV thickness

**Figure 4:**
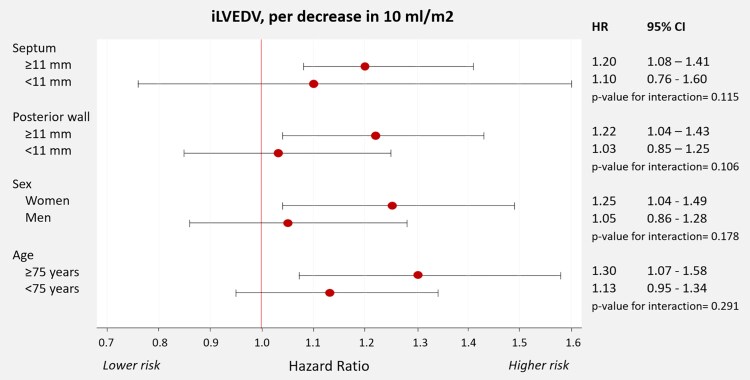
Association between iLVEDV and risk of incident HF in subjects with LVEF >60%. Subgroup analyses. Estimates of risk presented per decrease in 10 mL/m^2^. LVEF, left ventricular ejection fraction.

In subjects with LVEF > 60%, we found some numerical signs indicating a stronger association between smaller iLVEDV and higher risk of incident HF in women and subjects with greater LV thickness; however, the *P*-values for interactions were not statistically significant (*Figure 4*). We also did not find evidence for significant disparities across age (≥75 vs. < 75 years) (*Figure 4*).

### iLVEDV and other adverse clinical events

In the whole sample, iLVEDV was not independently associated with the risk of mortality, AMI, and non-related revascularization procedures (see Supplementary material online, *Figure S9*). We did not identify significant heterogeneous associations between iLVEDV and mortality, AMI, or revascularization procedures across LVEF categories (see Supplementary material online, *Figure S10*).

## Discussion

In a population of individuals with stage A–B HF (known or suspected CAD), iLVEDV, assessed by CMR, was associated with a U-shaped risk of incident symptomatic HF. Larger LV volumes and LVEF <50% identified those at increased risk. On the contrary, a heightened risk related to smaller ventricles was evident in individuals with LVEF >60% (Central illustration). This observation provides novel insights into LV structure and function relationships that separate distinctly different pathophysiological cardiac substrates predisposing to HF. The increased risk associated with supranormal LVEF was also paralleled when assessing standardized iLVEDV, stage A or B HF, and in individuals exhibiting a greater ratio of indexed LV mass to iLVEDV. To our knowledge, this is the first report to describe a significant association between smaller LV dimensions and long-term risk of developing symptomatic HF.

### HF: a heterogeneous syndrome

There is evidence that larger left ventricular size is associated with an increased risk of HF–related events, particularly in individuals with systolic dysfunction.^1^ This relationship is pathophysiologically supported by Laplace’s law, whereby increasing chamber radius augments wall stress, facilitating further ventricular dilation and worsening systolic function. However, the contribution of left ventricular size to the pathophysiology of HFpEF remains less well defined. HFpEF is a syndrome of high prevalence and significant morbidity and mortality, characterized by a complex and incompletely understood pathophysiology.^14^ A LVEF cut-off of ≥50% is commonly used to define HFpEF.^15^ However, recent studies suggest that this cut-off may not accurately differentiate HFpEF phenotypes, particularly in terms of pathophysiology and treatment response.^2,16,17^ There is increasing recognition that patients with LVEF > 60–65% are more frequently older women with stiffer ventricles showing a significant leftward shift in the end-diastolic pressure–volume relationship, making them more sensitive to preload and afterload changes.^16,17^ Patients with supranormal ejection fraction also do not appear to benefit from neurohormonal blockage.^18^

### Small left ventricle and supranormal ejection fraction: a marker of low cardiac reserve?

In the conventional understanding, larger LV dimensions are associated with a higher susceptibility to HF-related events and are generally regarded as an unfavourable structural trait.^1,19^ This concept is largely derived from population-based studies, such as the Multi-Ethnic Study of Atherosclerosis, demonstrating that LV dilation and eccentric remodelling are associated with an increased risk of incident HF, particularly in individuals with reduced or borderline systolic function.^19^ Consequently, progressive LV enlargement has traditionally been interpreted as a marker of adverse remodelling and decline in systolic function. However, in the absence of contractile dysfunction, the rules of displacement physics infer that the SVs can increase more with larger left ventricular volumes to allow for more vigorous myocardial systolic contractions.^20^ This adaptive form of LV enlargement is well recognized in endurance-trained athletes, who typically exhibit increased LV cavity size in parallel with higher SV and superior functional capacity as a consequence of exercise-induced cardiac remodelling.^21^

On the contrary, it is reasonable to assume that a lower LV chamber will result in a lower SV reserve. Along these lines and in agreement with current findings, a recent study by Foulkes et al. elegantly reported a reduced ability to enhance cardiac function during exercise among women with smaller cardiac dimensions.^2^ Specifically, peak VO2 was strongly inversely associated with CMR-assessed LV volumes in 185 healthy women. In this study, individuals with the largest LVEDV showed the greatest ability to reduce left ventricular end-systolic volume, allowing a larger SV increase.^2^ More recently, Rowe et al.^22^ found that small LV size independently predicts low cardiorespiratory fitness and functional disability, outperforming most echocardiographic parameters across diverse populations (endurance athletes, healthy non-athletes, individuals with unexplained dyspnoea, and individuals with HF with preserved ejection fraction). Our group has also confirmed an inverse relationship between iLVEDV and peak VO2 in two cohorts of patients with HFpEF.^23^ During normal aging, left ventricular volumes progressively decline.^24^ This phenomenon is consistent with the overrepresentation of older women with smaller cardiac dimensions among patients with HF and supranormal ejection fraction.^3^ The finding that smaller LV volumes are associated with a higher risk of incident HF was only found amongst those with supranormal ejection fraction and not in those with LVEF <60% is intriguing. In this sense, we postulate that an increased LVEF may identify subjects with low cardiac reserve (the inability to increase SV during exercise), where enhanced baseline contractility may be a compensatory mechanism to preserve cardiac output. In agreement with this postulate, Santas et al.^25^ have reported a strong and inverse association between LV size and LVEF in a large sample of patients with acute HF.

In agreement with this line of thought, Shah et al.^5^ reported in two large population-based cohort studies that a higher-than-normal LVEF was associated with adverse outcomes, in particular HF, among individuals with lower SV. More recently, in a large dataset of individuals with normal LVEF (*n* = 366 484), the authors found that small LV dimensions assessed by echocardiography were associated with increased mortality, especially in those with LVEF ≥ 60%.^26^ In HFpEF patients, Rosch et al.^16^ found differences in the haemodynamic response following isometric handgrip exercise across LVEF categories. Patients with an LVEF between 50 and 60% exhibited an increase in left ventricular filling pressure and end-diastolic volume, while patients with an LVEF above 60% had smaller ventricles and experienced an elevation in left ventricular filling pressure without a change in end-diastolic volume,^16^ an effect that may be attributable to the presence of diastolic tone.^27^ However, the assumption of higher LVEF as a proxy of increased myocardial contractility could be erroneous. An elegant study by Popovic et al. showed that patients with higher LVEF also exhibited systolic dysfunction, as assessed by alternative metrics of LV systolic function (preload recruitable stroke work and the ratio of LV stroke work to end-diastolic volume), which are parameters not influenced by diastolic stiffness or loading conditions and that consider chamber volume.^17^

It was also notable that the association between smaller LV volumes and the risk of HF in those with LVEF > 60% appears more pronounced in those with higher relative wall thickness, suggesting that LV concentric remodelling also plays a crucial role. Further studies are required.

### Potential clinical implications

The current findings and emerging evidence may have several clinical implications. First, assessment of LV size should be incorporated into the diagnostic workup for HF. In particular, the value of a small LV size as an additional diagnostic criterion for HFpEF warrants careful consideration.^28^ A consensus about the LV dimensions and sex-specific cut-offs for defining patients at risk of HFpEF is also warranted. Smaller LV dimensions may explain the higher prevalence and risk of women with supranormal ejection fraction.^29^ Second, this phenotypic presentation may benefit from differentiated treatment approaches. Eccentric remodelling might benefit those with smaller hearts and would favourably shift the end-diastolic pressure–volume relationship rightward to provide the larger systolic reserve.^17^ Aerobic exercise training can promote eccentric left ventricular remodelling,^30,31^ opening an exciting line of research for preventing and treating HFpEF. The feasibility and efficacy of different exercise-treatment approaches need to be defined. Likewise, a heart rate increase in this setting may improve cardiac output and eventually induce a limited degree of eccentric remodelling with LV enlargement.^32–34^ A recent *post hoc* analysis of the PRESERVE-HF randomized clinical trial, in which 52 ambulatory HFpEF, chronotropic incompetence, and stable treatment with β-blockers showed that lower iLVESV identified those with a greater short-term improvement peakVO2 after stopping β-blocker.^32^ Likewise, a substudy of the myPACE clinical trial involving preclinical and overt HFpEF with pre-existing pacemakers found that smaller LV volumes and higher LVEF identified patients who benefited most from personalized accelerated backup HR settings in terms of NT-proBNP reduction and improvement in pacemaker-detected daily activity levels at 1 year.^33^ Also, a subanalysis of this same last trial suggested that exposure to continuous accelerated pacing in HFpEF was associated with a reduced LV wall thickness and a small amount of LV dilation, with a small reduction in ejection fraction.^34^ Likewise, procedures like pericardial constraint alleviation have shown promise in increasing LV volumes.^35^ Lastly, and beyond the clinical perspective, these findings are particularly intriguing as they unveil a distinct pathophysiological mechanism for HFpEF.

### Limitations

The study, a retrospective analysis of Mediterranean patients with known or suspected CAD, has limitations that affect the generalizability of its findings. These limitations include (a) its inapplicability to healthy populations or different clinical scenarios, (b) lack of variables that could act as confounders, such as the absence of important HF risk factors (for instance, atrial fibrillation and renal function), comprehensive control of cardiovascular risk factors and pharmacological treatment, (c) reliance on EMR codes and diuretic treatment for HF diagnosis without assessment of natriuretic peptide or echocardiographic data in all patients. (d) CMR strain and diastolic assessment were not available precluding to examine the influence of a more accurate assessment of systolic and diastolic function on this analyses, and (e) LV volumes and function were assessed at rest without functional evaluation.

### Conclusions

This observational retrospective study suggested that smaller LV volumes and higher LVEF identified a subgroup of subjects with an increased risk of new-onset HF. Further studies are required.

## Supplementary Material

oeag009_Supplementary_Data

## Data Availability

The data that support the findings of this study are available from the corresponding author upon reasonable request, subject to institutional and ethical regulations.
