# Supplementary material for: Left ventricular diastolic volume on cardiac magnetic resonance and risk of incident heart failure
Source: Eur Heart J Open. 2026 Jan 24;6(1):oeag009. doi: 10.1093/ehjopen/oeag009 (PMC12915573; doi:10.1093/ehjopen/oeag009)
Supplement: oeag009_Supplementary_Data [file oeag009_supplementary_data.zip › Supplementary methods.docx]

**SUPPLEMENTARY METHODS**

**Vasodilator stress cardiovascular magnetic resonance (CMR) studies. Technical aspects**

All patients were examined with a 1.5 T system (Sonata Magnetom, Siemens, Erlangen, Germany) in our institution CMR laboratory according to the previously established study protocol^1^.

Analysis of images was performed by two cardiologists who are accredited by the European Society of Cardiology and have >15 years’ experience in the use and interpretation of vasodilator stress CMR testing. They performed and quantified in a core lab and in a single CMR facility located in our university hospital all outpatients CMR studies requested by health centers and outpatients’ clinics covered by our clinical department.

Cine images were acquired in 2-, 3-, 4-chamber views and in short-axis views using a steady state free precession sequence (repetition time/echo time: 25/1.6 ms, flip angle: 61º, matrix: 256 x 256, field of view: 320 x 270 mm, slice thickness: 7 mm).

Vasodilatation was induced with intravenous dipyridamole (0.84 mg/kg body weight over 6 minutes). After administering a gadolinium-based contrast agent at least 3 slices in the short-axis view and 1 section in the long-axis views were acquired for hyperemia first-pass perfusion imaging using a gradient-echo sequence (inversion time: 90 ms; effective repetition time/echo time: 182 ms/1 ms; flip angle: 12º; matrix: 192 × 96; field of view: 400 × 300 mm; slice thickness: 8 mm).

Late gadolinium enhancement imaging was performed 10 minutes after administering the gadolinium-based contrast agent in the same locations as in the cine images using a segmented inversion recovery steady-state free precession sequence (effective repetition time/echo time: 750 ms/1.26 ms; flip angle: 45º; matrix: 256 × 184; field of view: 340 × 235 mm; slice thickness: 7 mm). Inversion time was adjusted to nullify normal myocardium.

**Variability of measurements**

Inter-observer variability in calculating CMR indices was determined by comparing the differences between two measurements of the same case study performed separately by the two operators; thirty CMR studies randomly sampled from the registry were used for this purpose.

Intra-observer variability in calculating traditional CMR indices was determined by comparing the differences between two repeated measurements carried out by the operator with less experience (with an interval of one month from the first to the second measurement) in the thirty CMR studies from the registry used for calculation of inter-observer variability.

Inter- and intra-observer variability for the measurements of CMR (absolute and relative changes, coefficient of variation and intra-class correlation coefficients) are depicted in Online Tables 1A and 1B.

**Table 1A.** Inter-observer variability for CMR indices.

|  | **Relative change** | **Absolute change** | **Coefficient of variation** | **Intra-class correlation coefficient** |
| --- | --- | --- | --- | --- |
| **LVEF** | 4±3% | 2±1% | 0.208 | 0.989 |
| **Indexed LV end-diastolic volume** | 7±4% | 5±4 ml/m^2^ | 0.301 | 0.981 |
| **Indexed LV end-systolic volume** | 5±5% | 2±2 ml/m^2^ | 0.508 | 0.994 |
| **Ischemic burden** | 5±5% | 0.5±1 segments | 1.37 | 0.997 |
| **LGE** | 4±5% | 0.4±1 segments | 1.70 | 0.998 |

CMR: cardiovascular magnetic resonance; LGE: late gadolinium enhancement; LV: left ventricle; LVEF: left ventricular ejection fraction.

**Table 1B.** Intra-observer variability for CMR indices.

|  | **Relative change** | **Absolute change** | **Coefficient of variation** | **Intra-class correlation coefficient** |
| --- | --- | --- | --- | --- |
| **LVEF** | 3±2% | 1.5±0.7% | 0.204 | 0.994 |
| **Indexed LV end-diastolic volume** | 5±3% | 3±2 ml/m^2^ | 0.293 | 0.994 |
| **Indexed LV end-systolic volume** | 4±5% | 1±2 ml/m^2^ | 0.504 | 0.996 |
| **Ischemic burden** | 3±4% | 0.3±1 segments | 1.45 | 0.995 |
| **LGE** | 3±2% | 0±1 segments | 1.82 | 0.998 |

CMR: cardiovascular magnetic resonance; LGE: late gadolinium enhancement; LV: left ventricle; LVEF: left ventricular ejection fraction.

**REFERENCES**

1. Bodi V, Husser O, Sanchis J, et al. Prognostic implications of dipyridamole cardiac MR imaging: a prospective multicenter registry. *Radiology*. 2012;262:91–100.
