# Supplementary material for: Left ventricular diastolic volume on cardiac magnetic resonance and risk of incident heart failure
Source: Eur Heart J Open. 2026 Jan 24;6(1):oeag009. doi: 10.1093/ehjopen/oeag009 (PMC12915573; doi:10.1093/ehjopen/oeag009)
Supplement: oeag009_Supplementary_Data [file oeag009_supplementary_data.zip › Supplementary Table 1.docx]

**Supplementary Table 1.** Estimates of risk of covariates included in the multivariate Cox regression analysis for predicting new-onset HF accounting for competing events

|  | **HR (CI 95%)** | **p-value** |
| --- | --- | --- |
| Age, per 1 year increase | 1.05 (1.04-1.07) | <0.001 |
| Women | 1.64 (1.20-2.25) | 0.002 |
| Hypertension | 1.12 (0.81-1.55) | 0.489 |
| Diabetes Mellitus | 1.95 (1.47-2.59) | <0.001 |
| Dyslipidemia | 0.94 (0.70-1.12) | 0.649 |
| Family history of IHD | 1.72 (0.91-3.27) | 0.096 |
| Smoking status | 0.86 (0.55-1.36) | 0.519 |
| Ischemic heart disease | 0.74 (0.52-1.12) | 0.213 |
| Lef bundle branch block | 0.85 (0.48-1.49) | 0.557 |
| Prior coronary revascularization | 1.51 (1.04-2.20) | 0.044 |
| Ability to perform an exercise stress test | 0.53 (0.33-0.88) | 0.014 |
| Septum thickness, per increase in 1 mm | 1.01 (0.95-1.07) | 0.757 |
| Posterior wall thickness, per increase un 1 mm | 1.08 (1.0-1.17) | 0.050 |
| CMR perfusion deficit, per increase in 1 segment | 1.03 (0.98-1.09) | 0.258 |
| CMR transmural late gadolinium, per increase in 1 segment | 0.95 (0.85-1.04) | 0.266 |
| Related revascularization | 1.25 (0.78-2.02) | 0.351 |

CMR: cardiovascular magnetic resonance; HF: heart faiure; IHD: ischemic heart disease

Competing events: All-cause mortality, MI, and non-related CMR revascularization during follow-up were considered competing events

Harrell’s C statistic of the model: 0.80
