# Supplementary figures and images for: Left ventricular diastolic volume on cardiac magnetic resonance and risk of incident heart failure
Source: Eur Heart J Open. 2026 Jan 24;6(1):oeag009. doi: 10.1093/ehjopen/oeag009 (PMC12915573; doi:10.1093/ehjopen/oeag009)

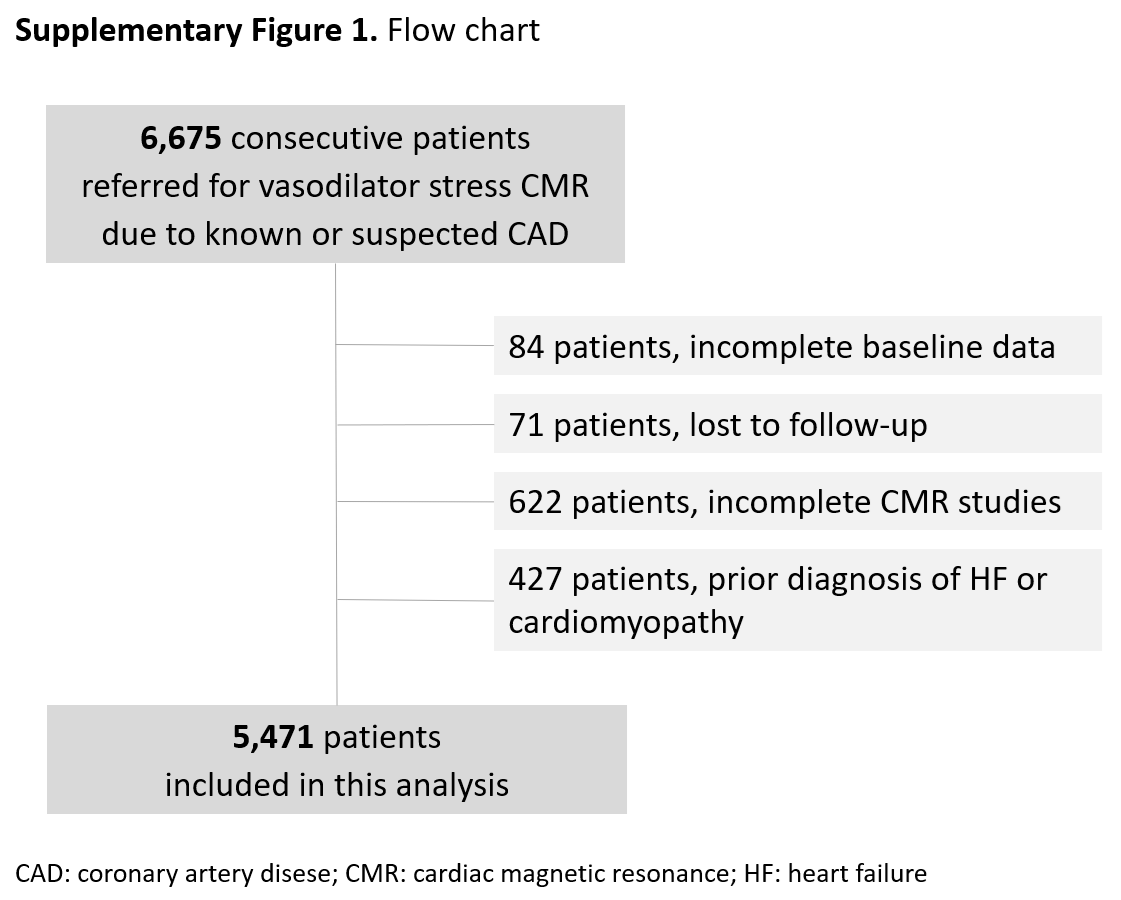

Supplement: oeag009_Supplementary_Data [file oeag009_supplementary_data.zip › Figure S1.tif]

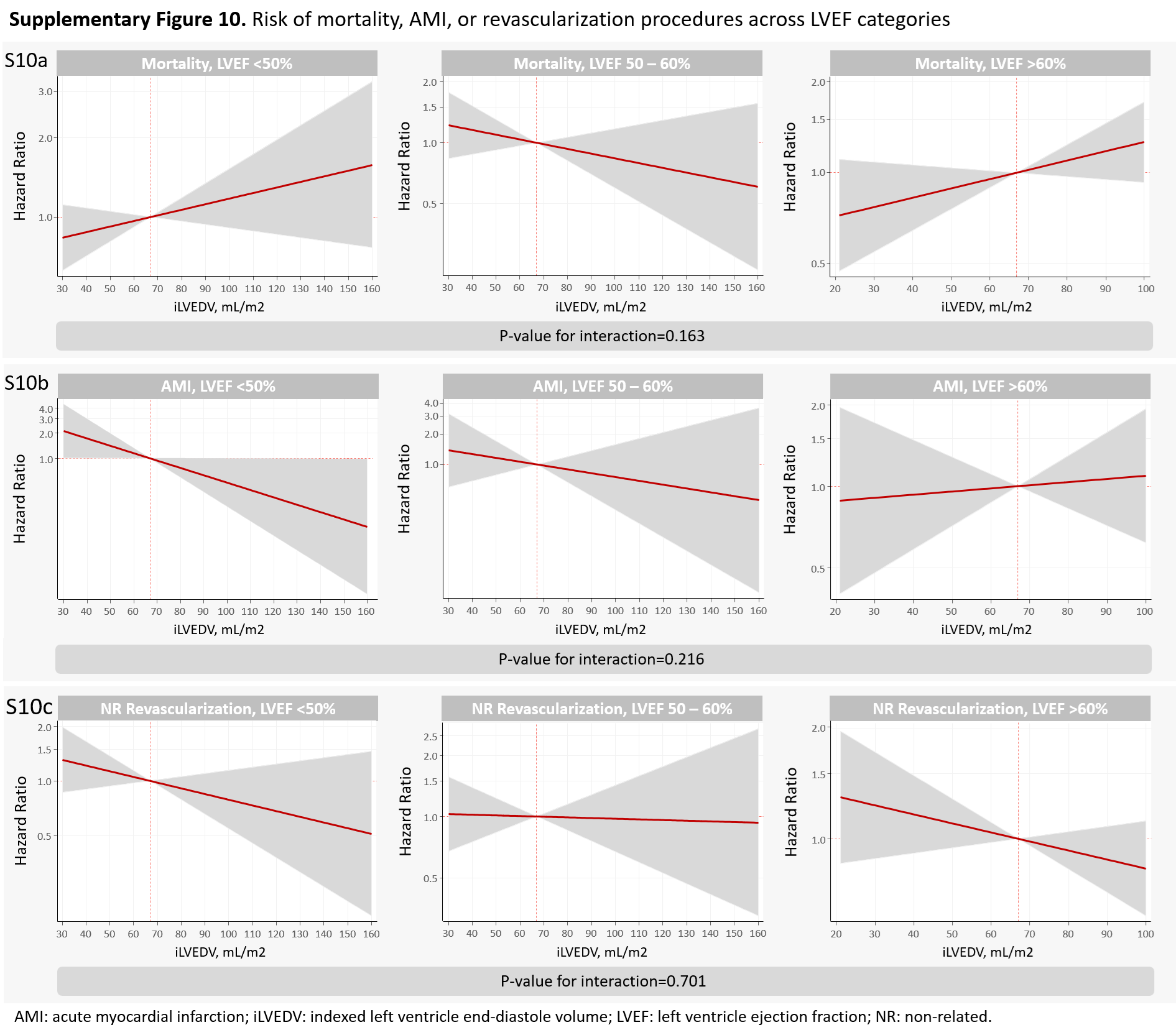

Supplement: oeag009_Supplementary_Data [file oeag009_supplementary_data.zip › Figure S10.tif]

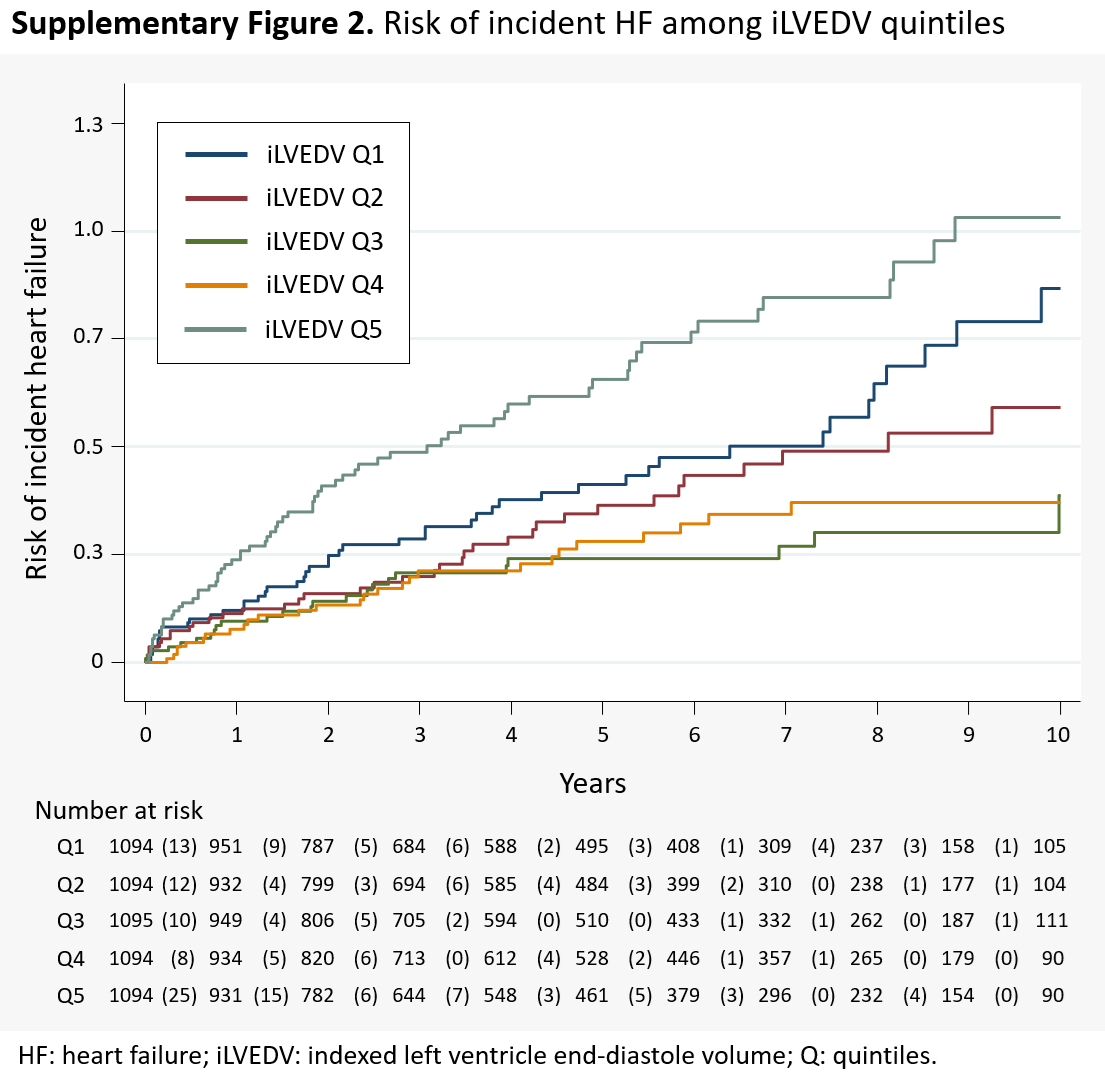

Supplement: oeag009_Supplementary_Data [file oeag009_supplementary_data.zip › Figure S2.tif]

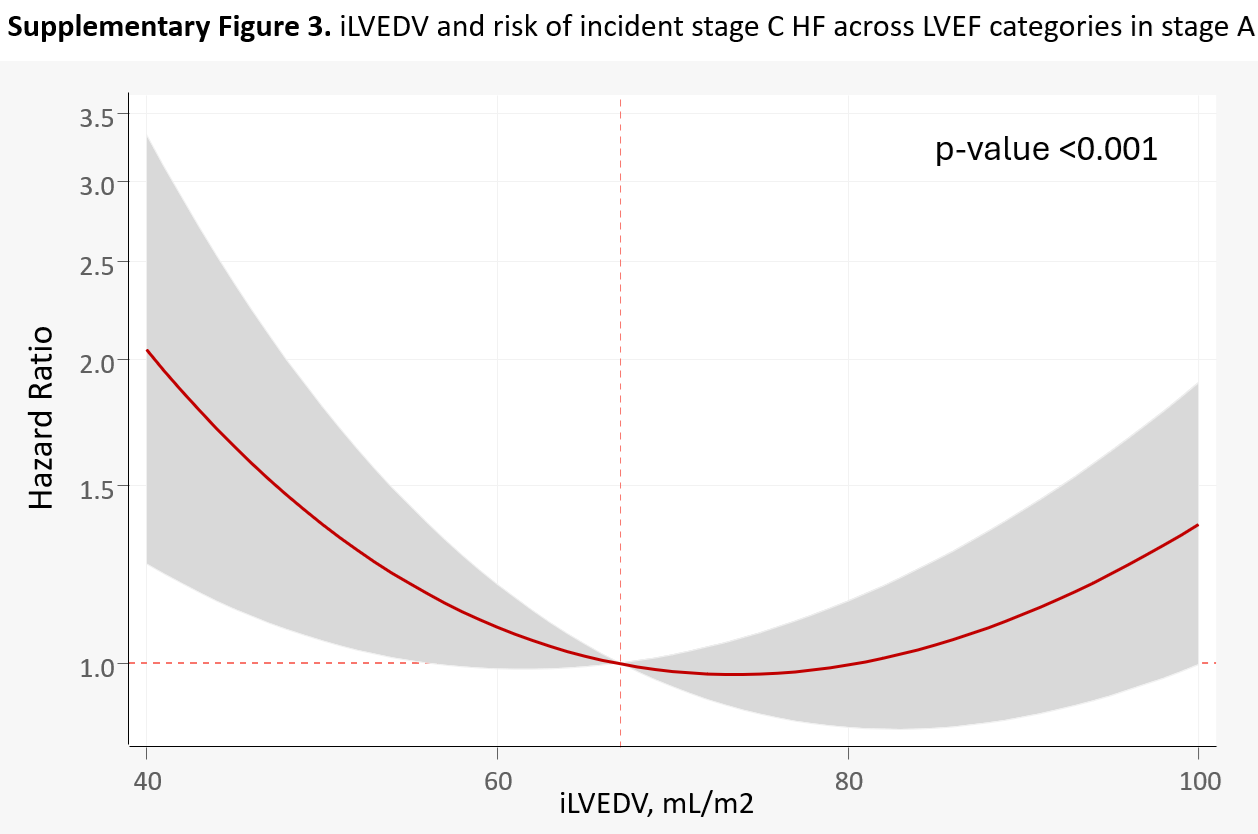

Supplement: oeag009_Supplementary_Data [file oeag009_supplementary_data.zip › Figure S3.tif]

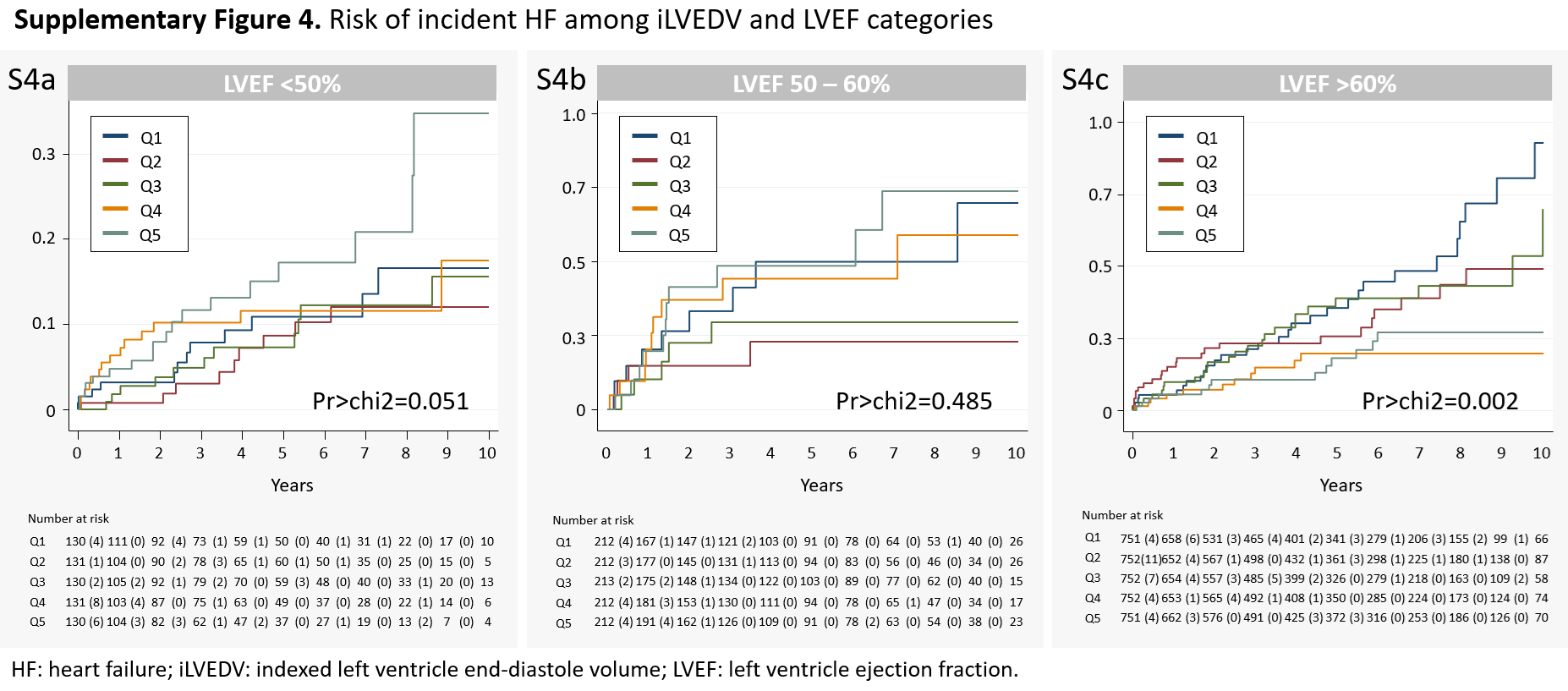

Supplement: oeag009_Supplementary_Data [file oeag009_supplementary_data.zip › Figure S4.tif]

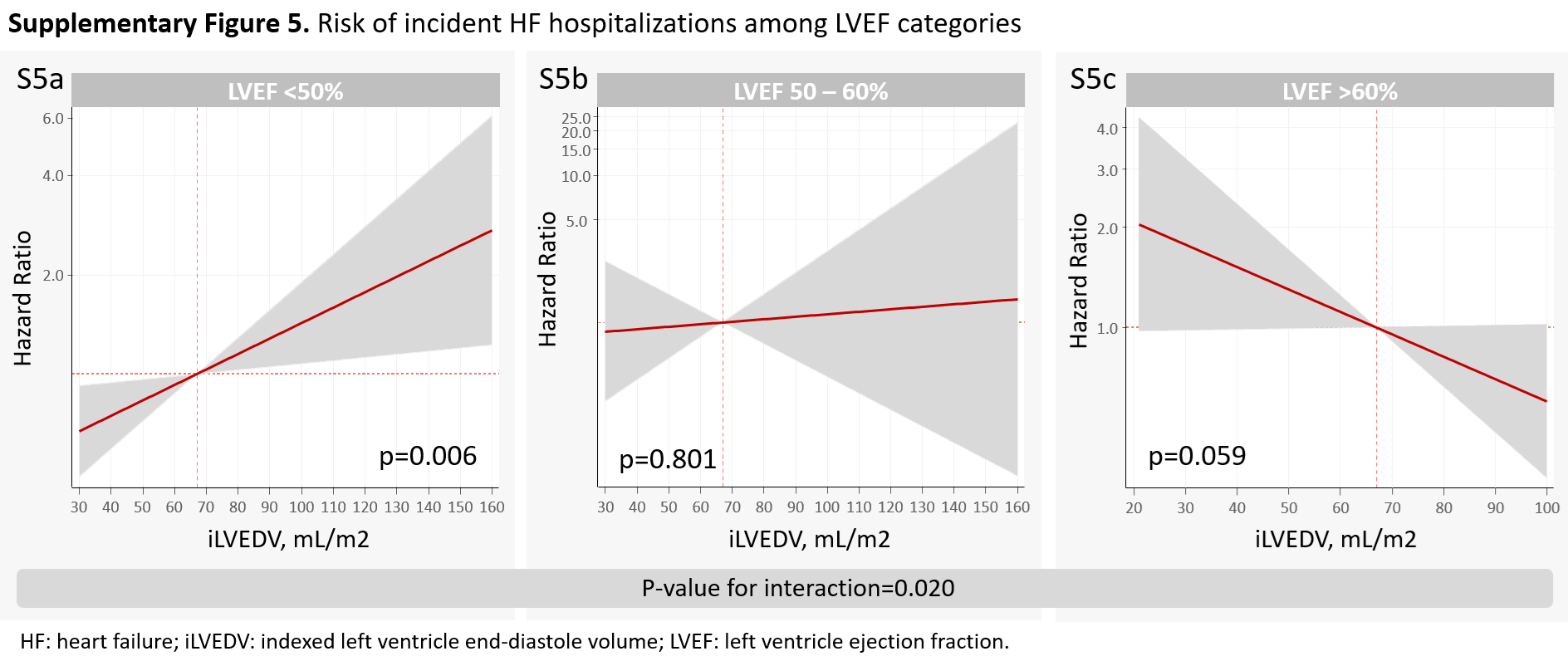

Supplement: oeag009_Supplementary_Data [file oeag009_supplementary_data.zip › Figure S5.tif]

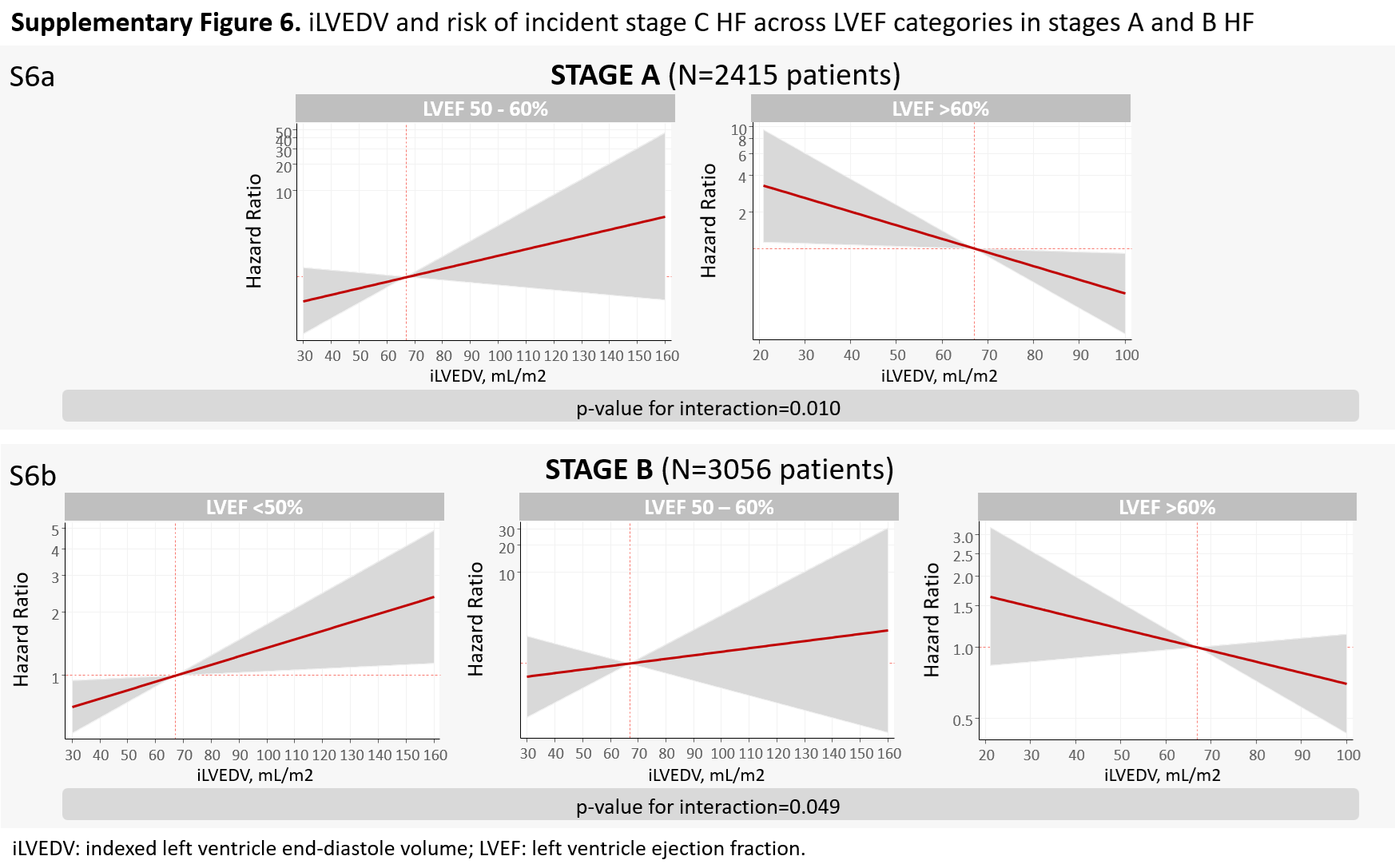

Supplement: oeag009_Supplementary_Data [file oeag009_supplementary_data.zip › Figure S6.tif]

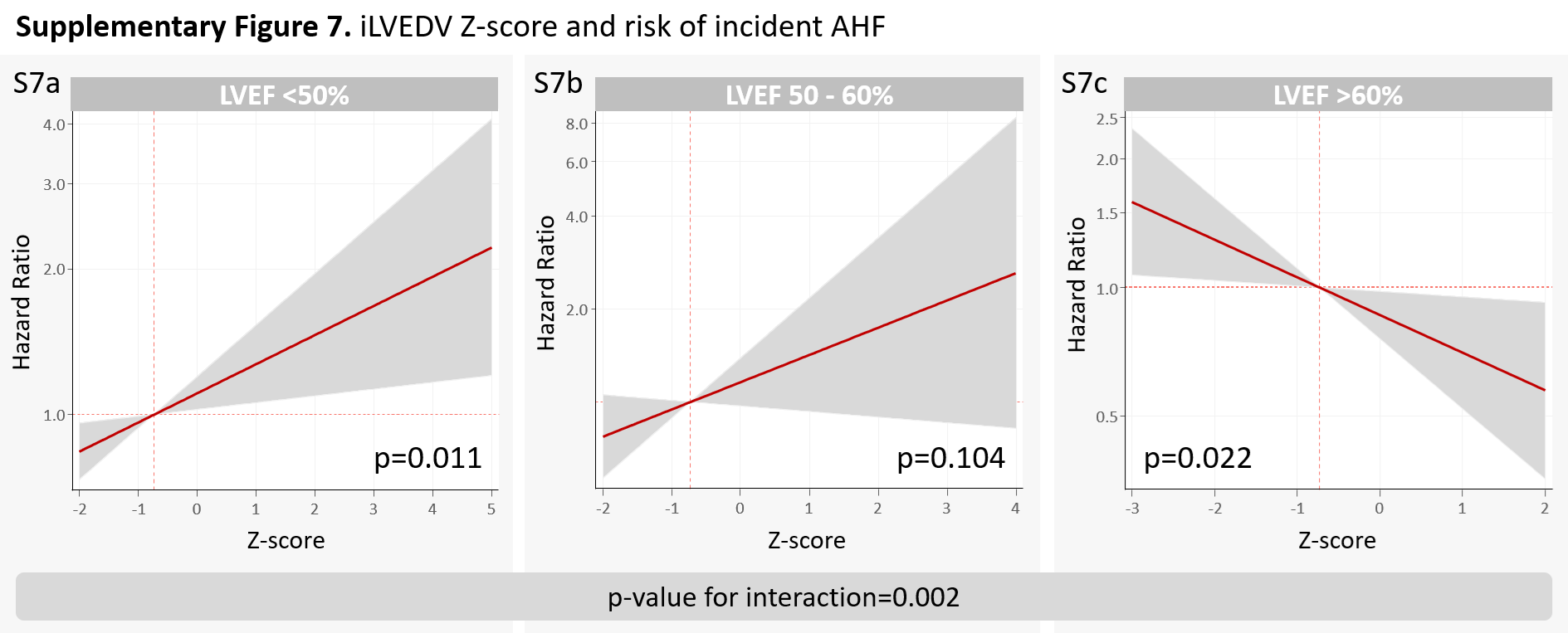

Supplement: oeag009_Supplementary_Data [file oeag009_supplementary_data.zip › Figure S7.tif]

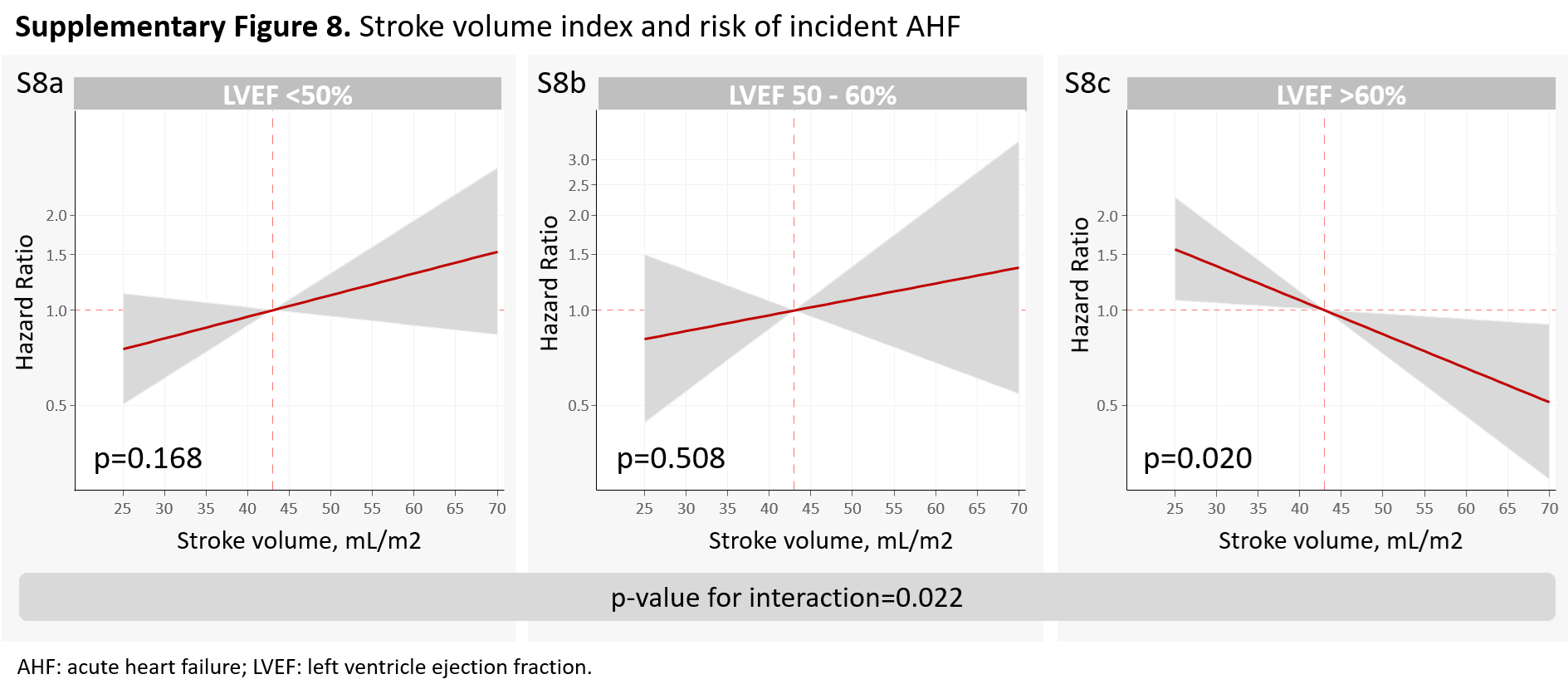

Supplement: oeag009_Supplementary_Data [file oeag009_supplementary_data.zip › Figure S8.tif]

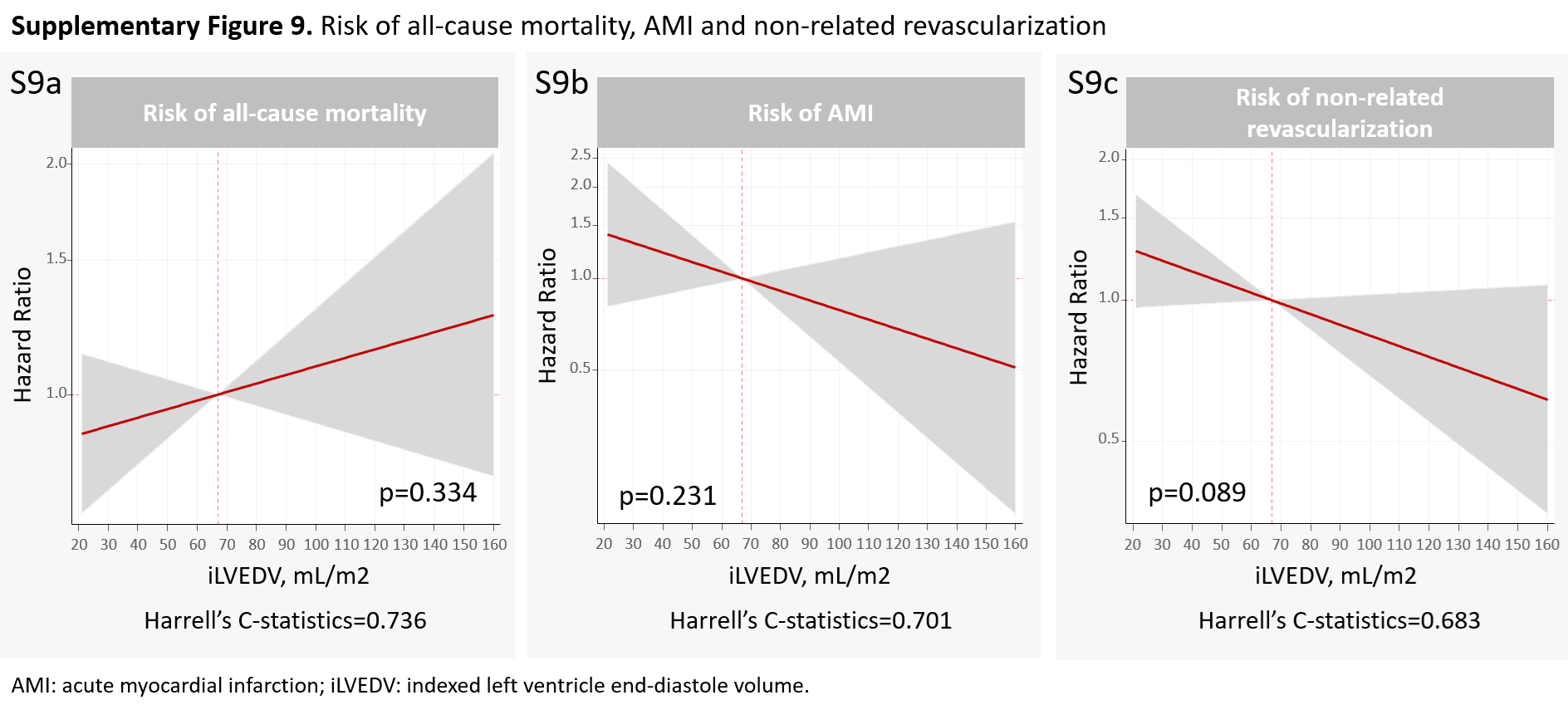

Supplement: oeag009_Supplementary_Data [file oeag009_supplementary_data.zip › Figure S9.tif]
